# Endovascular retrieval of bone cement entrapped in an inferior vena cava filter after percutaneous vertebroplasty: a case report

**DOI:** 10.3389/fsurg.2026.1913450

**Published:** 2026-09-29

**Authors:** Guangxu Wan, Jingwei Yan, Qichen Liang, Ziteng Zhang, Ziqiang Sun, Peng Liu

**Affiliations:** 1 Department of Vascular Surgery, Affiliated Hospital of Jining Medical University, Jining, Shandong, China; 2 School of Clinical Medicine, Jining Medical University, Jining, Shandong, China; 3 Department of Thoracic Surgery, Affiliated Hospital of Jining Medical University, Jining, Shandong, China; 4 Department of Thoracic Surgery, Qinghai Red Cross Hospital, Xining, Qinghai, China

**Keywords:** bone cement leakage, case report, endovascular foreign body retrieval, inferior vena cava filter, percutaneous vertebroplasty

## Abstract

Percutaneous vertebroplasty (PVP) is widely used to treat osteoporotic vertebral compression fractures, however, bone cement leakage may enter the venous system and cause potentially serious embolic complications. Entrapment of migrated bone cement within an inferior vena cava filter (IVCF) is rare and may pose substantial technical challenges during filter retrieval. We report the case of a 67-year-old woman with bilateral deep vein thrombosis who underwent placement of a Denali retrievable IVCF after thrombus progression while receiving a nonstandard initial rivaroxaban regimen selected because of concern about bleeding risk. She subsequently underwent PVP for osteoporotic thoracolumbar compression fractures and returned 5 months after filter placement for elective retrieval. Pre-retrieval ultrasonography showed a patent inferior vena cava (IVC) but did not identify the intrafilter abnormality. Intraoperative inferior vena cavography revealed an obvious filling defect within the IVCF. A radiopaque shadow on fluoroscopy and retrospective review of prior PVP images suggested an impacted bone cement foreign body. Conventional retrieval through the right internal jugular vein failed. After reassessment of the procedural risks and discussion with the patient and her family, a 22F large-bore sheath was introduced through the femoral vein, and a temporary protective IVCF was deployed in the infrarenal IVC, caudal to the renal vein ostia and cephalad to the mobilized Denali filter–cement complex. The Denali retrievable IVCF and entrapped bone cement were retrieved *en bloc* through femoral venous access, and the protective filter was subsequently removed using the LOOP technique. The patient remained hemodynamically stable and had no fever, chest tightness, dyspnea, or abdominal pain. Follow-up computed tomography showed no hematoma, IVC stenosis, or IVC occlusion, and she was discharged in stable condition. This case highlights that, in patients with a history of PVP and an indwelling IVCF, intrafilter bone cement entrapment should be considered before planned filter retrieval. Careful review of previous fluoroscopic or interventional images, preoperative abdominal radiography followed by selective IVC CT venography (CTV) when radiopaque material is suspected, routine intraoperative cavography, preparation of large-bore sheaths and protective filters, and readiness for open surgical conversion may improve procedural safety.

## 1 Introduction

Percutaneous vertebroplasty (PVP) is an effective minimally invasive treatment for osteoporotic vertebral compression fractures and can rapidly relieve pain and enhance vertebral stability. Bone cement leakage is one of the most common complications of PVP, with reported rates ranging from approximately 0.9% to 26% (1). Most cement leakages are clinically asymptomatic. However, when cement enters the paravertebral venous plexus, it may migrate through the azygos system or other valveless venous communications into the caval system and subsequently to the pulmonary arteries, causing asymptomatic or symptomatic pulmonary embolism (2).

Inferior vena cava filters (IVCFs) are designed to intercept thromboemboli from the lower extremities, but their lattice may also capture solid intravascular foreign material. Bone cement entrapment within an IVCF has been described only in isolated reports, with management ranging from observation to endovascular removal or open cavotomy, and no standardized retrieval strategy exists (3, 4). We describe a technically distinct case in which standard jugular retrieval failed and the filter–cement complex was removed *en bloc* through a 22F femoral sheath after deployment of a second, infrarenal protective filter.

## 2 Case report

### 2.1 Patient information and chronological clinical course

The patient was a 67-year-old woman with type 2 diabetes mellitus, systemic lupus erythematosus, Sjögren’s syndrome, coronary atherosclerotic heart disease managed with coronary artery stenting, osteoporosis, long-term oral glucocorticoid use, a history of thrombocytopenia and recurrent cutaneous ecchymoses, and prior vertebral compression fractures treated with percutaneous vertebroplasty (PVP) at T12, L2, and L3.

At the initial presentation, venous ultrasonography at another hospital showed bilateral peroneal-vein thrombosis. Although she had no absolute contraindication to anticoagulation, long-term oral glucocorticoid use, a history of thrombocytopenia, and recurrent cutaneous ecchymoses raised concern about bleeding, we therefore initiated rivaroxaban at 20 mg once daily without the recommended initial regimen of 15 mg twice daily for 21 days. Four days later, repeat ultrasonography at our hospital showed extension to the bilateral femoral, popliteal, and peroneal veins, together with thrombosis of calf muscular veins; the D-dimer concentration was 8.00 μg/mL on the following day. Given imaging-documented thrombus progression during this nonstandard initial regimen, six days after the initial diagnosis a Denali retrievable IVCF (DL950F; BD/Bard, Tempe, AZ, USA) was implanted therapeutically through the right femoral vein. The filter apex was placed in the infrarenal IVC, and rivaroxaban 20 mg once daily was continued after discharge (Figures 1, 2A).

**Figure 1:**
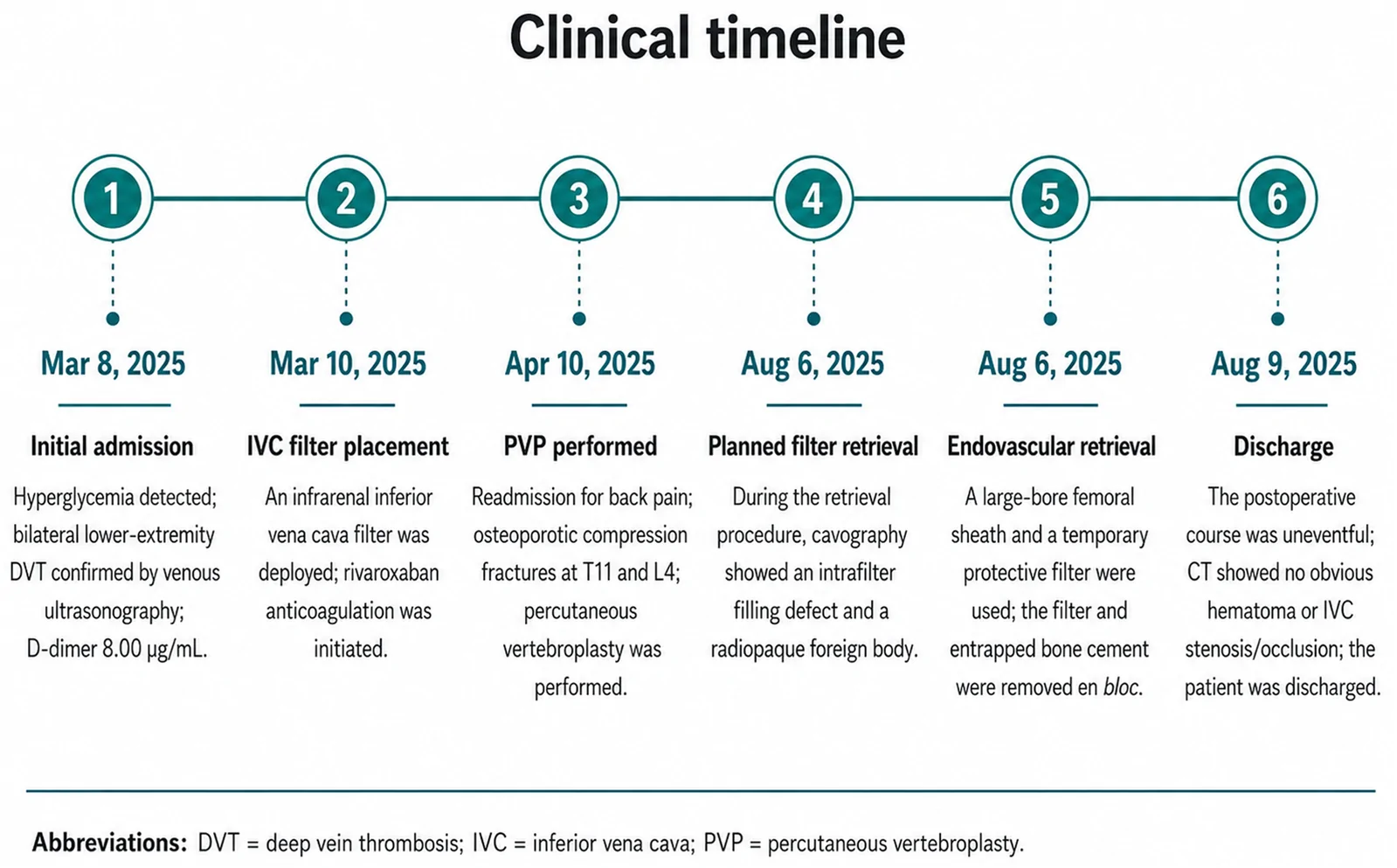
Clinical timeline of the patient.

**Figure 2:**
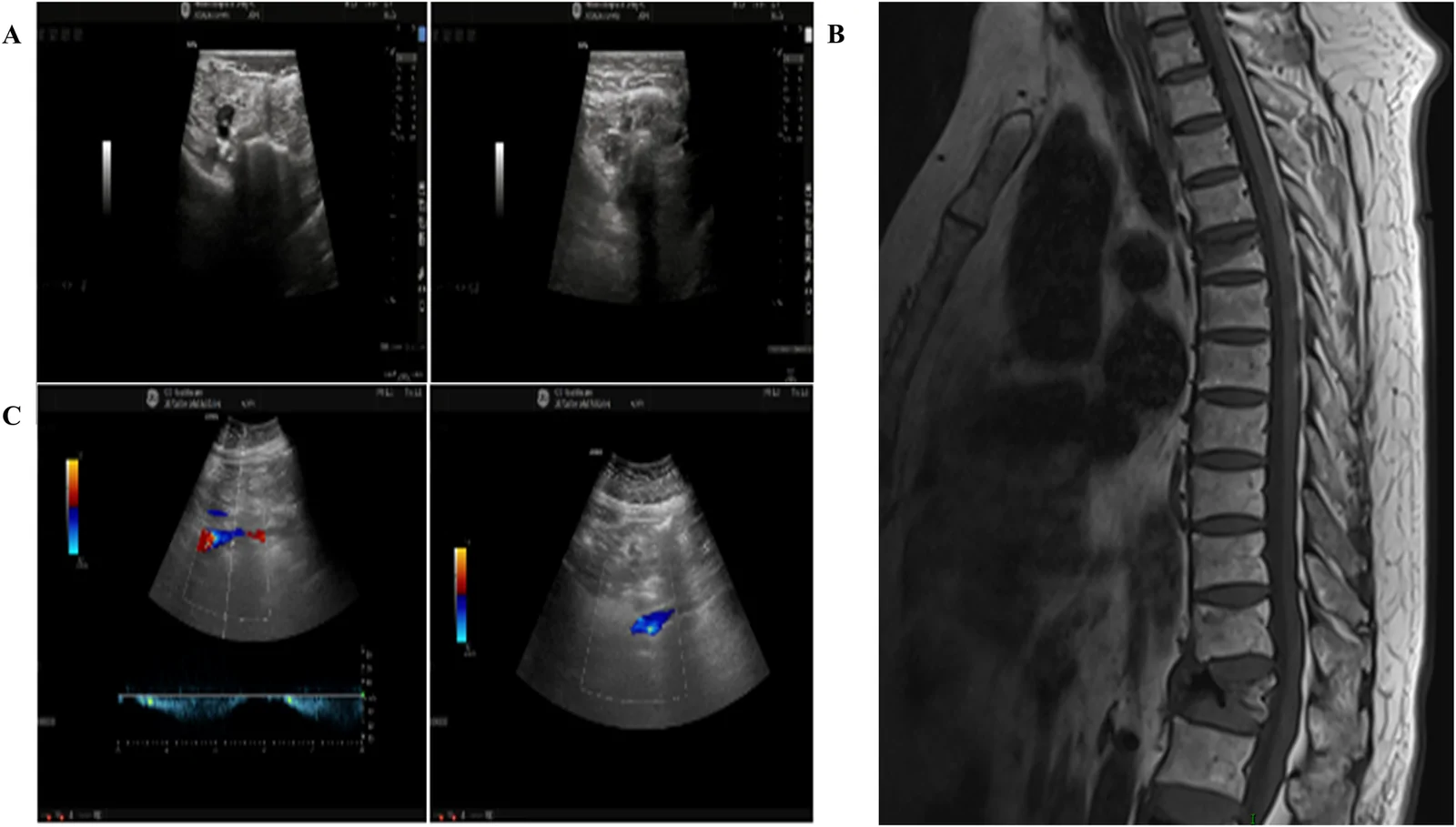
Pre-retrieval clinical imaging. **(A)** Lower-extremity venous ultrasonography demonstrated bilateral DVT. **(B)** Spinal MRI demonstrated osteoporotic vertebral compression fractures. **(C)** Color Doppler ultrasonography demonstrated the indwelling IVCF within a patent infrarenal IVC.

Approximately 1 month after IVCF placement, the patient was readmitted because of back pain and was diagnosed with thoracolumbar osteoporotic pathological fractures involving the T11 and L4 vertebral bodies (Figure 2B). After surgical contraindications were excluded, percutaneous vertebroplasty was performed on the same day. The post-PVP clinical course was uneventful.

Approximately 5 months after IVCF placement, follow-up venous ultrasonography showed post-IVCF implantation changes. The IVC wall was not thickened, the lumen diameter was normal, and a filter-like strong echo was visible within the lumen. Color Doppler imaging showed a patent lumen without an obvious abnormal flow signal or a definite intrafilter abnormality (Figure 2C). Two days later, the patient was admitted for elective IVCF retrieval. Her temperature was 36.7 °C, pulse rate was 69 beats/min, respiratory rate was 17 breaths/min, and blood pressure was 152/106 mmHg. Her height was 156 cm and body weight was 69 kg. Both lower limbs had normal skin color and temperature, without clinically evident edema or superficial venous dilation; distal perfusion was preserved and the dorsalis pedis pulses were palpable bilaterally.

### 2.2 Therapeutic intervention

At our institution, routine pre-retrieval assessment had consisted of lower-extremity and IVC ultrasonography plus D-dimer testing, with cavography planned at retrieval. Preoperative IVC CT venography (CTV) was not obtained in this case because of cost considerations and the expectation that intraoperative cavography would define IVC patency. In retrospect, this approach was inadequate because ultrasonography may confirm IVC patency but may fail to detect radiopaque cement within the filter. A dedicated abdominal radiograph before retrieval can provide a low-cost screening examination for radiopaque material within or adjacent to the IVCF. If a suspicious radiopaque structure is identified, or if clinical suspicion remains despite an inconclusive radiograph, IVC CTV should be performed to define the size, morphology, craniocaudal extent, and relationship of the cement to the filter struts and caval wall, as well as associated thrombus and venous patency. When CTV is performed, unenhanced CT images, if available, can confirm the intrinsic high attenuation of the cement and complement the venous-phase images; however, routine dual-phase CT is not required for every patient. If imaging demonstrates extensive or irregular cement incorporation that appears unsuitable for endovascular retrieval, the procedural risks and alternative strategies, including long-term filter retention or open surgical removal, should be discussed with the patient and family before retrieval is attempted.

During the retrieval procedure, cavography was performed under local infiltration anesthesia with anteroposterior fluoroscopy. From the jugular access, fluoroscopy showed a radiopaque structure within the Denali filter, and cavography demonstrated a patent IVC with a corresponding intrafilter filling defect. Retrospective review of the PVP fluoroscopic images showed venous cement migration toward the filter, making entrapped bone cement the leading diagnosis (Figure 3).

**Figure 3:**
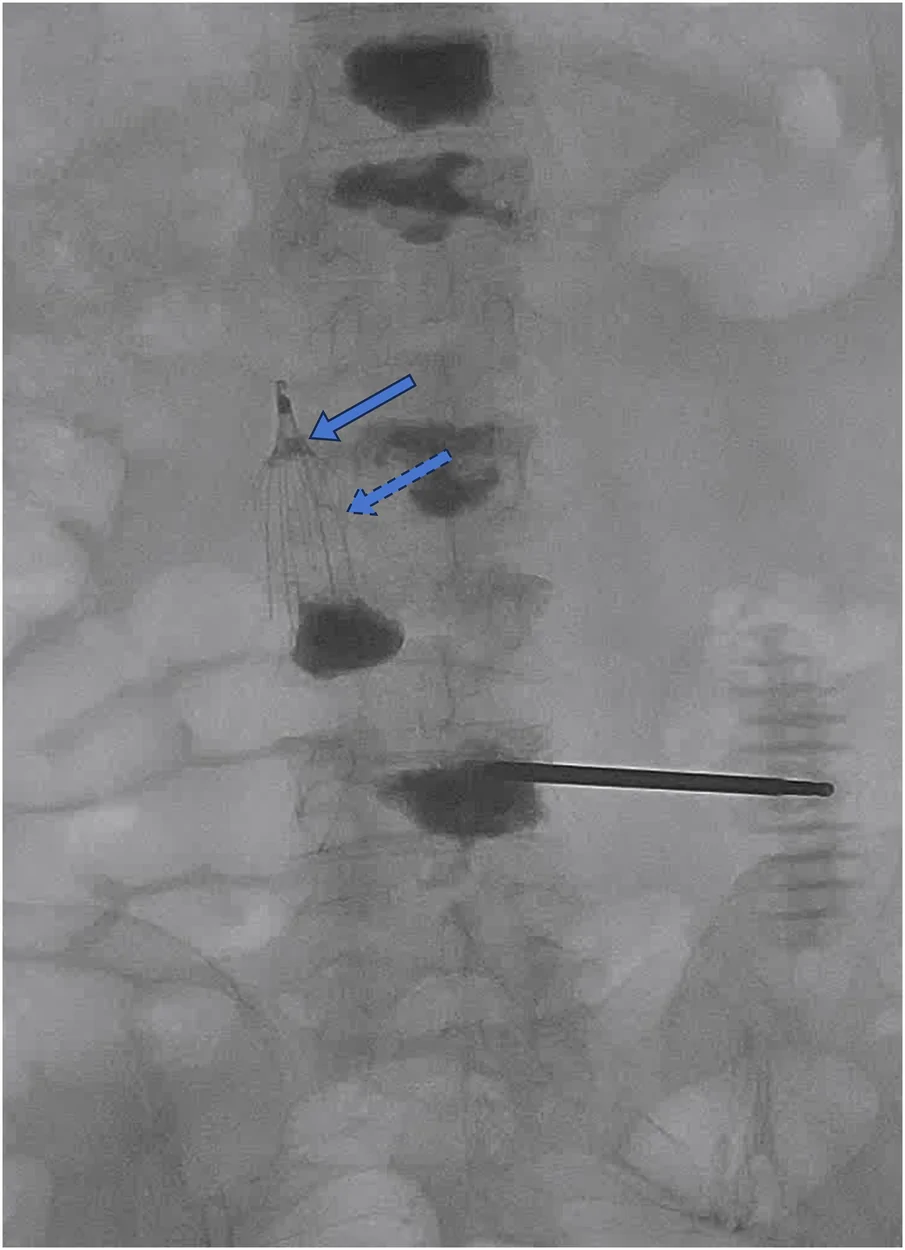
Intraoperative anteroposterior fluoroscopy and cavography showing the denali IVCF (open arrow), radiopaque intrafilter cement (solid arrow).

A standard jugular snare engaged the Denali retrieval hook, and the filter partially entered the retrieval sheath. The jugular approach was abandoned when the irregular cement could not enter the sheath and tended to move caudally during traction. Further force was judged to create an unacceptable risk of cement separation, free IVC migration, right-heart embolization, or pulmonary cement embolism. The procedure was paused, and the operative strategy and potential need for open surgical conversion were reassessed with a senior vascular surgeon. The procedure was subsequently completed endovascularly without conversion to open surgery.

A 22F, 33-cm hydrophilic GORE DrySeal Flex introducer sheath (DSF2233; W. L. Gore & Associates, Flagstaff, AZ, USA) was then advanced through the right femoral vein. Direct grasping of the cement with an IVCF retrieval set (RK1901; Zylox-Tonbridge Medical Technology, Hangzhou, China) was unsuccessful. The wire-loop maneuver was performed stepwise as follows. First, the tip of a 5F, 100-cm angiographic catheter (451-503H5; compatible with a 0.035-inch guidewire) was trimmed into a hook shape. Second, the catheter was passed around the cranial aspect of the filter. Third, a 0.035-inch, 260-cm hydrophilic guidewire (RF*GA35263M; Terumo, Tokyo, Japan) was advanced approximately 10 cm beyond the catheter. Fourth, the distal end of the guidewire was captured with the RK1901 device through the right femoral sheath and externalized, creating a loop around the filter–cement complex. Fifth, controlled traction was applied to the two externalized guidewire ends to draw the encircled complex toward the 22F sheath. In this case, the Denali filter and cement moved together, became engaged at the mouth of the sheath, and shifted caudally rather than entering the sheath completely. Because simultaneous mobilization of the filter and cement created a risk of cement fragmentation, caval migration, and pulmonary embolization, further traction was suspended. Before further traction, the left femoral vein was accessed and an Aegisy retrievable IVCF (XJLX3260; LifeTech Scientific, Shenzhen, China) was deployed in the infrarenal IVC, caudal to the renal vein ostia and cephalad to the mobilized Denali filter–cement complex. The protective filter was not placed suprarenally.

With the infrarenal protective filter positioned cephalad to the mobilized filter–cement complex, the Denali retrievable IVCF and impacted cement were withdrawn *en bloc* through the right femoral 22F sheath. The retrieved filter was intact, and gross inspection showed a hard, irregular, radiopaque fragment (Figure 4). No histopathologic, spectroscopic, or formal material analysis was performed; therefore, the diagnosis was based on gross appearance, fluoroscopy, cavography, and retrospective PVP imaging. Repeat venography showed a patent IVC and iliac veins without extravasation. The Aegisy protective filter could not be captured with standard snaring and was removed through the left femoral access using a loop-snare technique. Final venography again confirmed patency without vascular injury. Total procedure duration was 152 min, fluoroscopy time was 15 min 35 s, and the kerma-area product (KAP) was 35.58 Gy·cm². Estimated blood loss was approximately 100 mL.

**Figure 4:**
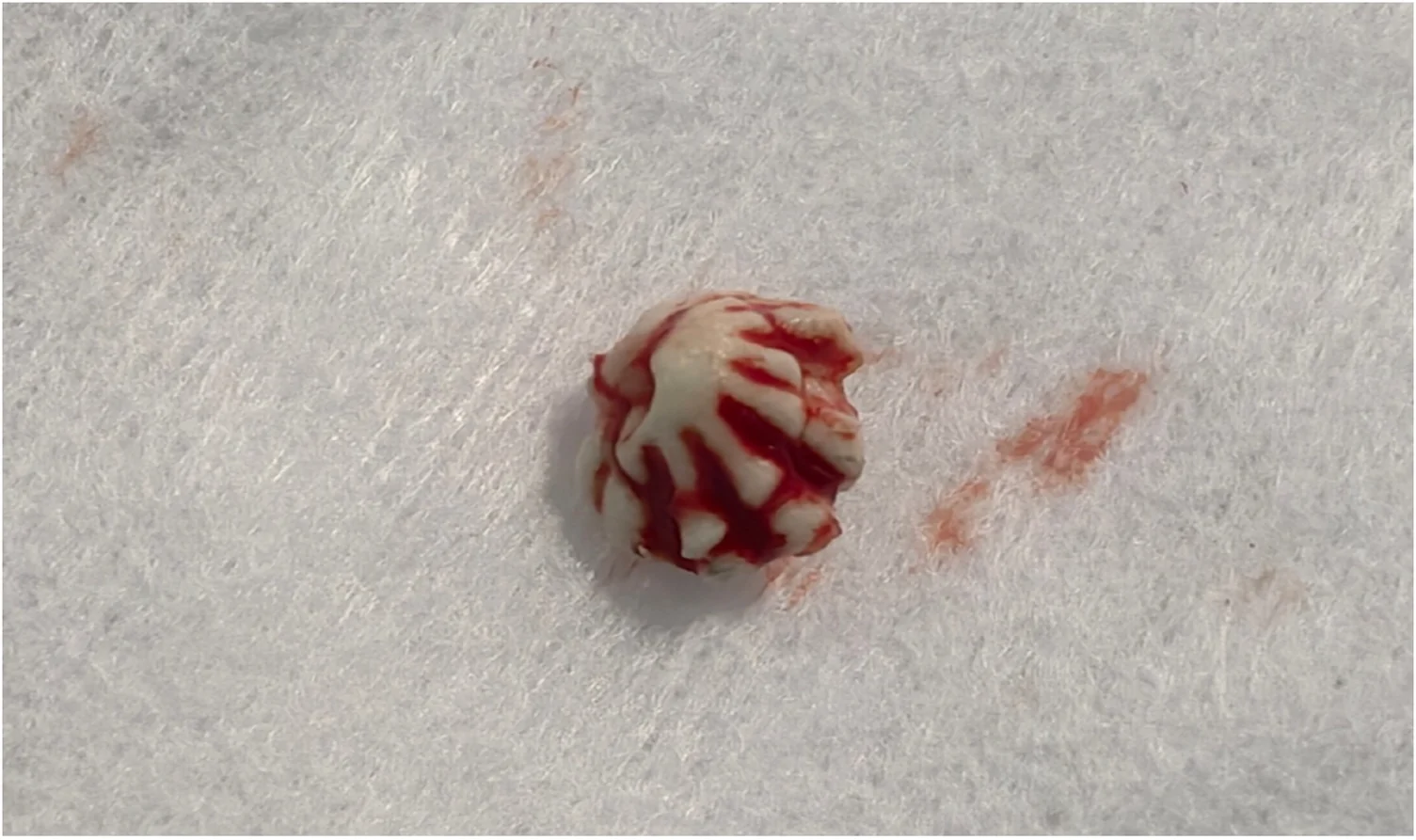
Retrieved specimen.

All access sheaths and devices were removed after final venography. The puncture sites were treated with compression dressings; management of the right large-bore femoral access is detailed below.

### 2.3 Follow-up and outcomes

After the procedure, the patient had no fever, chest tightness, dyspnea, or abdominal pain. Mild pain in the bilateral groin regions and lower back gradually resolved. On postoperative day 1, the D-dimer level was 0.49 *μ*g/mL, and coagulation parameters showed no obvious abnormalities. Abdominal and pelvic computed tomography showed no definite bleeding, hematoma formation, or inferior vena cava stenosis or occlusion.

Because a large-bore sheath had been used in the right groin, oozing occurred at the puncture site after decompression. Hemostasis was achieved with simple interrupted suturing of the subcutaneous tissue and skin, followed by repeated compression. The patient remained clinically stable and was discharged on postoperative day 3. Rivaroxaban 15 mg once daily was continued for 2 months and then discontinued. At 2-month clinical follow-up, she reported no lower-extremity pain or swelling, palpitations, chest tightness, or dyspnea.

## 3 Discussion

The unique feature of this case is that the bone cement that leaked and migrated after PVP did not enter the pulmonary arteries but was instead trapped and impacted within a previously implanted IVCF. This course indicates that, in addition to capturing thrombi, an IVCF may provide protection against large solid foreign bodies within the venous system. However, when the trapped foreign body is hard and irregular in shape, planned filter retrieval may be transformed from a routine procedure into a complex endovascular foreign body retrieval (5–7).

The recommended rivaroxaban treatment regimen for patients with acute DVT is 15 mg twice daily with meals for the first 21 days, followed by 20 mg once daily with meals. However, this patient had been receiving long-term oral glucocorticoid therapy and had a history of thrombocytopenia and recurrent skin ecchymoses. Although these factors are not absolute contraindications for anticoagulation therapy, they suggest that the patient may be at a higher risk of clinically significant bleeding; therefore, there were significant concerns about implementing the standard initial intensive anticoagulation regimen. After weighing the risk of continued thrombus progression against the anticipated risk of bleeding, the treatment team did not adopt the standard initial regimen of 15 mg twice daily for 21 days but instead administered rivaroxaban 20 mg once daily. This approach should be understood as an individualized adjustment based on the patient’s specific bleeding risk, rather than a universally recommended alternative. The subsequent proximal progression of the thrombus suggests that the intensity of anticoagulation during the acute phase may have been insufficient. However, a causal relationship between the two cannot be established based on a single case. This case highlights that when the standard initial anticoagulation regimen is deemed potentially unsafe, early clinical and imaging reassessments should be intensified, and alternative anticoagulation strategies should be considered.

During PVP, pressurized polymethylmethacrylate can escape from vertebral trabeculae into the basivertebral veins and anterior external vertebral venous plexus. From lumbar levels, cement may pass through the lumbar or ascending lumbar veins into the IVC, whereas thoracic pathways may communicate with the azygos or hemiazygos system before reaching the central venous circulation. In this patient, the exact route could not be proven, but retrospective PVP fluoroscopy showed venous cement migration toward the infrarenal filter, making entry through the vertebral-lumbar venous network the most plausible pathway. Once within the IVC, a cement fragment larger than the spaces between the filter struts, or one with an irregular branching configuration, can become mechanically interlocked in the filter rather than continue to the right heart and pulmonary arteries. The filter may therefore avert pulmonary cement embolization while simultaneously creating a complex filter-foreign body unit that is hazardous to retrieve.

The occult nature of cement leakage is another important lesson from this case. Pre-retrieval ultrasonography showed a patent IVC and a filter-like strong echo but did not identify the intrafilter abnormality. Intraoperative fluoroscopy and cavography subsequently demonstrated a radiopaque structure and corresponding filling defect within the filter, and the diagnosis was supported by the gross appearance of the retrieved fragment and retrospective review of the PVP images. Therefore, in patients with a history of PVP and an indwelling IVCF, ultrasonography alone may be insufficient to exclude a high-attenuation foreign body within the filter.

Published cases confirm that management must be individualized. In the six cases summarized by Han et al., cemented filters were either retained, removed endovascularly or by an incompletely described approach, or removed by open cavotomy. Table 1 summarizes cement location, filter type, retrieval or management strategy, reported complications, and clinical outcomes or follow-up, together with the present case. Information unavailable in the original reports is explicitly designated as “Not reported” and was not inferred.

**Table 1:** Previously reported cases of bone cement trapped by an inferior vena cava filter and comparison with the present case.

Report	Cement location	Filter type	Retrieval strategy	Complications	Outcomes
Herbstreit et al., 2006 (6)	Cement extending into the IVC and entrapped within the filter	Not reported	Open cavotomy	Not reported	Filter/cement removed
Athreya et al., 2009 (8)	IVC cement cast after PVP; filter placed above the cast	Not reported	Gooseneck snare to right common femoral vein, followed by surgical removal	Not reported	Cement removed; recovery reported
Agko et al., 2010 (7)	IVC cement fragment after kyphoplasty; filter placed for protection	Greenfield	Endovascular mobilization to common femoral vein, followed by open venotomy	Transient hypotension; retroperitoneal exploration found no vascular injury	Cement removed; asymptomatic at 4 weeks
Li et al., 2013 (9)	Cement captured during PVP	OptEase	Conservative management	No filter-related complication at 8 months	Filter retained
Edwards et al., 2015 (10)	PMMA trapped after spinal revision	Not reported	Difficult endovascular filter-removal procedure; details incompletely reported	Not reported	Filter removal reported
Prater et al., 2021 (11)	Cement within lumbar vein, IVC, and a pre-existing IVCF after vertebroplasty	Not reported	Nonoperative management after treatment options were discussed	Not reported	Filter retained
Present case	Fragment entrapped in Denali filter after PVP	Denali DL950F; protective Aegisy XJLX3260	Failed jugular retrieval; 22F femoral sheath; infrarenal protection; *en bloc* extraction	Controllable right-groin bleeding; no IVC injury or clinical PE	Both filters and fragment removed

IVC, inferior vena cava; IVCF, inferior vena cava filter; PMMA, polymethylmethacrylate; PVP, percutaneous vertebroplasty.

From the perspective of preoperative assessment, in patients with a history of PVP, percutaneous kyphoplasty, or other procedures that may lead to intravascular foreign body migration, previous intraoperative fluoroscopic images and postoperative imaging data should be systematically reviewed. Abdominal radiography may be used as an initial screening examination because radiopaque cement can appear as an abnormal linear or irregular opacity within the filter region. When radiography demonstrates suspicious material, or when clinical suspicion remains despite equivocal radiographic findings, IVC CT venography (CTV) should be obtained to determine the filter position, degree of tilt, wall apposition, presence of thrombus, size and morphology of the cement, its relationship to the filter struts and caval wall, and patency of the IVC and iliac veins. If unenhanced CT images are acquired, they can confirm the intrinsic high attenuation of the cement, whereas the venous phase provides complementary information regarding vascular anatomy, thrombus, and luminal patency. This staged imaging strategy helps determine the necessity, feasibility, and potential risks of filter retrieval (12, 13). If the cement is extensive, markedly irregular, or firmly incorporated into the filter or caval wall and is considered unsuitable for endovascular retrieval, long-term filter retention or open surgical removal should be discussed with the patient and family.

The operative strategy in this case emphasized the principle of protection before retrieval. Conventional filter retrieval sheaths have a limited diameter and cannot accommodate an irregular cement fragment impacted within the filter. Forceful retrieval through the internal jugular approach may cause the cement to dislodge from the filter, resulting in iatrogenic pulmonary embolism. After conversion to a large-bore femoral sheath, the route was shorter and the direction of manipulation was more direct, which facilitated *en bloc* advancement of the filter and cement into the sheath. Meanwhile, deployment of a temporary IVCF in the infrarenal IVC, caudal to the renal vein ostia and cephalad to the mobilized filter–cement complex, provided secondary embolic protection in case of foreign body dislodgement and was a key measure to reduce the risk of intraoperative pulmonary embolism.

Device preparation also defines the safety margin of the procedure. Intraoperative review suggested that the bone cement was hard and unlikely to fragment under ordinary compression or cutting. However, its irregular shape and impaction within the filter basket may still cause retrieval difficulty, filter deformation, or vascular wall injury. Therefore, vascular sheaths of different diameters and lengths, snares, grasping devices, instruments required for the wire-loop or loop-snare technique, temporary filters, and vascular closure or compression devices should be prepared in advance. The maneuver used in this case was a case-specific application of an established loop-snare concept rather than a novel retrieval technique. Because the loop simultaneously encircled the filter and impacted cement, traction could mobilize both structures together and carried risks of cement fragmentation, filter deformation, caval injury, and embolization. Accordingly, traction should be controlled and immediately suspended if the complex cannot be safely advanced into the sheath, with proximal embolic protection and open surgical conversion available. In this case, the 22F large-bore sheath provided a channel for *en bloc* retrieval, but sheath length and support may still affect procedural stability. If available, a sheath with a more appropriate length and stronger support may further reduce technical difficulty (14).

Perioperative management is also important because the use of a large-bore femoral venous sheath increases the risk of puncture-site bleeding and subcutaneous hematoma. In this case, oozing occurred at the right groin puncture site after compression was released and was controlled by local suturing and repeated compression. This experience suggests that, when a large-bore femoral sheath is used for retrieval of a filter and foreign body, careful puncture-site planning, hemostatic strategy after sheath removal, and postoperative compression and observation are essential. When necessary, preplaced closure devices, figure-of-eight sutures, subcutaneous sutures, or internal Z-sutures may be used to reinforce hemostasis, and a venography catheter should be retained before sheath removal to assess the integrity of the access vessel and inferior vena cava.

This case also has several limitations. First, it cannot establish the general safety, efficacy, or reproducibility of the described retrieval strategy. Second, preoperative inferior vena cava CTV was not performed, and intrafilter bone cement impaction was not identified before surgery. Third, no histopathologic, spectroscopic, or other formal material analysis was performed; therefore, identification of the retrieved fragment as bone cement was based on its gross appearance, radiopacity, intraoperative imaging, and retrospective review of the PVP images. Fourth, postoperative imaging was limited to abdominal and pelvic CT obtained during the index hospitalization, whereas the 2-month follow-up was clinical only. Consequently, delayed IVC stenosis, thrombosis, or other procedure-related changes could not be assessed radiographically. Fifth, wall apposition of the protective filter increased the difficulty of its subsequent retrieval, while the limited length and support of the large-bore sheath reduced procedural stability. Finally, puncture-site management could be further improved. These limitations provide useful considerations for the management of similar cases in the future.

## 4 Conclusion

Bone cement can become firmly interlocked with an indwelling IVCF and convert routine retrieval into a high-risk foreign-body procedure. After vertebral cement augmentation in a patient with an IVCF, pre-retrieval abdominal radiography should be considered even when ultrasonography is unremarkable; a suspicious or equivocal radiographic finding should prompt IVC CTV for further characterization. If imaging demonstrates extensive or irregular cement incorporation that appears unsuitable for endovascular retrieval, long-term filter retention or open surgical removal should be considered through shared decision-making. If standard jugular retrieval fails because the cement cannot be safely sheathed, a planned bailout using large-bore femoral access, a second infrarenal filter placed cephalad to the complex, and *en bloc* extraction may be feasible. This strategy should be undertaken only with complete device preparation, multidisciplinary risk reassessment, and immediate availability of open surgical conversion.

## Data Availability

The raw data supporting the conclusions of this article will be made available by the authors, without undue reservation.
